# Alcohol consumption and breast cancer-specific and all-cause mortality in women diagnosed with breast cancer at the New York site of the Breast Cancer Family Registry

**DOI:** 10.1371/journal.pone.0189118

**Published:** 2017-12-15

**Authors:** Nur Zeinomar, Ashley Thai, Ann J. Cloud, Jasmine A. McDonald, Yuyan Liao, Mary Beth Terry

**Affiliations:** 1 Department of Epidemiology, Mailman School of Public Health, Columbia University, New York, NY, United States of America; 2 Herbert Irving Comprehensive Cancer Center, Columbia University, New York, NY, United States of America; Ohio State University Wexner Medical Center, UNITED STATES

## Abstract

**Purpose:**

Alcohol consumption is an established and important risk factor for breast cancer incidence in the general population. However, the relationship between alcohol and mortality among women with breast cancer is less clear. This study examines the effect of alcohol consumption on mortality in women affected with breast cancer at baseline from a high-risk family breast and ovarian cancer registry.

**Methods:**

We studied 1116 women affected with breast cancer at baseline from the Metropolitan New York Registry. The examined reported alcohol consumption (total of beer, wine, liquor) was defined as the average number of drinks per week reported from age 12 to age at baseline. We assessed vital status of each participant using participant or family reported data and we used the National Death Index to supplement deaths reported through family updates. We used Cox proportional hazards models to estimate the association between alcohol intake and overall mortality (HR^**O**^), breast cancer-specific mortality (HR^**BC**^), and non-breast cancer mortality (HR^NBC^), adjusted for confounders.

**Results:**

After a mean follow-up of 9.1 years, we observed 211 total deaths and 58 breast cancer deaths. Compared to non-drinkers, we found that both low and moderate to heavy levels of alcohol intake were not associated with greater overall mortality (≤3 drinks/week: HR^O^: 0.66, 95% CI: 0.38–1.14); > 3 drinks/week: HR^O^: 1.16, 95% CI: 0.85–1.58), breast cancer–specific mortality (≤ 3 drinks/week: HR^BC^:0.62, 95% CI: 0.19–2.03; >3 drinks/week: HR ^BC^: 0.96, 95% CI: 0.49–1.89), or non-breast cancer-specific mortality (≤3 drinks/week: HR ^NBC^: 0.73, 95% CI: 0.32–1.6; >3 drinks/week: HR^NBC^: 1.18, 95% CI: 0.75–1.86).

**Conclusions:**

Alcohol intake reported from age 12 to age at baseline was not associated with overall or breast cancer-specific mortality in this cohort of affected women with a family history of breast cancer.

## Introduction

Alcohol consumption is associated with an increased risk of various cancers including oral, pharyngeal, esophageal, liver, colon and breast cancer[1]. Specifically for breast cancer, a modest increase in risk (30%-50%) among women with moderate alcohol consumption has been consistently observed in many epidemiological studies [2–5]. However, studies on the association between alcohol consumption and mortality among those with a history of breast cancer have been less consistent.

Three studies, including two large prospective cohorts have supported an association with alcohol intake and overall breast-cancer mortality [6–8]. In contrast, three prospective cohort studies by Harris et al. (2012) (n = 3,146), Newcomb et al. (2013) (n = 22,890), and Barnett et al. (2008) (n = 4,560) found that post-diagnostic alcohol was not associated with breast cancer mortality [9–11]. One study by Kwan (n = 1,897) showed that post-diagnostic intake of ≥6 g/day was associated with an elevated but not statistically significant breast cancer mortality rate (HR = 1.52, 95%CI 1.00–2.29) [12]. Other studies examined cumulative alcohol intake, including a cross sectional study by Breslow (n = 184,764) and a prospective cohort by Holm (n = 1,212) and found no association between alcohol intake and breast cancer mortality [13, 14]. There is also evidence that alcohol consumption may be associated with decreased breast cancer mortality. Two prospective cohort studies by Hellmann (n = 528) and Fuchs (n = 85,709) found that 0.1–14.9g/day of alcohol intake was associated with a 4–33% decrease in the rate of breast cancer mortality [15, 16]. However, at a higher intake of >15 g/day, risk of death from breast cancer increased by 37–67%[15].

Whether moderate alcohol consumption is harmful in those with a breast cancer diagnosis is important for women with extensive breast cancer family history where the risk and benefits may be less clear. Most studies only consider family history as a confounder in the association between alcohol and mortality [6, 7, 10, 11, 15]. One exception found no difference between alcohol intake and breast cancer-specific mortality in women with a family history [7]. Understanding whether alcohol intake is associated with mortality after breast cancer diagnosis in women with a breast cancer family history is important as alcohol consumption is a common and modifiable risk factor.

Because modest alcohol intake has been associated with reduced cardiovascular risk[15], and women are more likely to die of cardiovascular disease even after breast cancer diagnosis[11], the perceived benefits of alcohol on overall mortality in women may be driven by reduction in heart disease mortality, despite increases in other cause mortality such as breast cancer[15]. Given the lack of consistency between alcohol intake and breast cancer-specific mortality, and lack of data on the relation between alcohol intake and mortality in women with a family history[17], we conducted a prospective cohort study using data from the New York site of the Breast Cancer Family Registry (BCFR) in which we collected detailed information on alcohol consumption in women at high risk for breast cancer. We investigated the association between alcohol consumption from age 12 until age at baseline and overall mortality, breast cancer-specific, and non-breast cancer mortality in women affected with breast cancer at baseline and explored differences across Hispanic/non-Hispanic ethnicity, age, body mass index (BMI), and estrogen/progesterone hormone receptor status.

## Materials and methods

### Study population

The BCFR is an ongoing prospective cohort of adult men and women with a family history of breast or ovarian cancer established in 1995 (for more information see [18]). It includes six research sites in the USA, Canada, and Australia that ascertain families either from cancer registries (identifying population-based families) or seen in clinical and community settings (identifying clinic-based families). For this study, we included participants from the New York site of the BCFR (The Metropolitan NY Registry) a clinic-based site that identified participants along with family members based on the following criteria: (1) a female with either breast or ovarian cancer age 45 or less at diagnosis; (2) a male with a history of breast cancer; (3) a female with a history of both breast and ovarian cancer; (4) a female with one first-degree relative or two second-degree relatives with a history of either breast or ovarian cancer; (5) a family with a known BRCA1 or BRCA2 mutation. In terms of follow-up, we contacted at least one participant from each family annually to update personal and family cancer histories and deaths, as well as some exposures addressed in the core epidemiology questionnaire.

The New York site enrolled 4004 individuals, of which 1139 women were affected with breast cancer at baseline and eligible for this analysis. These women were enrolled into the study between 1996 and 2011, with over 75% enrolled by 2001. We included women who completed a baseline questionnaire and for whom alcohol consumption information was available. We excluded 7 participants with missing baseline alcohol information and 16 participants who did not provide information on the amount of alcohol consumed during different age ranges. The final sample of 1116 women included 9.5% women with carcinoma in situ and 90.5% women with invasive breast cancer. The median time between diagnosis and study entry for women included in the study was 4.4 years. A pathologist reviewed pathology reports, estrogen and progesterone (ER/PR) hormone receptor status reports, histological slides and/or paraffin tumor blocks from the treating institution were used to confirm diagnoses [18]. Cases with missing ER/PR status were more likely to be cases diagnosed before 1996 when collection of ER/PR information was less routine at community hospitals. All participants provided informed written consent and Columbia University’s Institutional Review Board approved this protocol.

### Definition of mortality

We obtained participant or family reported vital status during follow-up. For participants whose updated vital status was missing from family reported data, we used the National Death Index (NDI) to supplement the missing data. The following ICD codes were used for breast cancer-specific deaths: ICD-9 174 or ICD-10 C50. In the final analyses, individuals lost to follow-up and sent to NDI for matching were only considered deceased if scores assigned by the NDI met cut-offs for an accurate match[19]. We assessed sensitivity of NDI matching for outcome measures, in which we compared family reported deaths to NDI reported deaths. Overall, the registry accounted for a total of 201 overall deaths, 33 of which were breast cancer deaths. Linkage to NDI added four breast cancer deaths and six other deaths, for a total of 211 deaths. Additionally, 21 deaths in the registry with unknown cause of death were linked to NDI as breast cancer death, which brings the total number of breast cancer deaths in the study to 58.

### Definition of alcohol consumption

From questionnaires at baseline, we collected information on alcohol consumption including type of alcohol consumed: beer, wine/wine coolers and hard liquor. Women who answered “no” to ever consuming any alcoholic beverages regularly (at least once per week for 6 months or longer) were classified as non-drinkers. Women who answered “yes” to regularly consuming alcoholic beverages were classified as drinkers and were asked to separately report their average of number of drinks of beer, wine/wine coolers, and hard liquor consumed per week, for the following age periods: 12–17, 18–24, 25–34, 35–44, 45–54 and ≥55 years [18]. One drink was defined as a 12 oz. serving of beer, one medium glass of wine/wine cooler, or one shot of liquor [18]. We calculated alcohol intake for each age period as the sum of the intake of each of the three different types of alcoholic beverages consumed (beer, wine, liquor). To examine the average alcohol intake prior to diagnosis, we applied weights to each age period where the weights were equivalent to the number of years spent in the age interval.

Given the way that alcohol intake information was reported (in age periods, not exact ages), we could not accurately parse out pre- and post-diagnostic alcohol consumption. As such, our definition of alcohol intake represents long-term exposure over the life course that includes pre-diagnosis and may include a time-period post-diagnosis. However, previous reports, including one that specifically examined ductal carcinoma in-situ, have noted that there was little substantial change in alcohol consumption patterns following a cancer diagnosis [10, 11, 20–22]. To minimize misclassification of alcohol intake and ensure that sample size was sufficient for adjusted statistical analyses, three categories of intake were used; nondrinkers, low intake (≤3 drinks per week) and moderate to heavy intake (>3 drinks per week), analogous to a US previous study [13]. We conducted sensitivity analyses to ensure that effect did not differ between those who consumed 3–6 drinks/week and those who consumed ≥7 drinks/week.

### Statistical analysis

We used Cox proportional hazards models to estimate adjusted hazard ratios (HR) and their 95% confidence intervals (95% CI) for association of alcohol intake and mortality (overall, breast cancer-specific mortality, and non-breast cancer). We calculated person time from the start of follow-up (the time of questionnaire return) until the earliest of the following events: death, loss to follow-up, or end of follow-up (December 31, 2012). A total of 165 women of 1116 were lost to follow-up. We used a robust variance estimator to account for the family structure of the cohort. We adjusted all models for age at baseline, defined as the age of enrollment into the study. We tested models for confounding by commonly accepted breast cancer risk factors: ethnicity (non-Hispanic white, Hispanic, other), parity (centered at nulliparous), age at first live birth (centered at mean age), age at menarche (<12, 12–13, and >13 years), age at menopause (centered at mean age), history of HRT use (ever, never), oral contraceptive use (ever, never), smoking status (ever, never), education (< high school, high school or vocational tech, college or graduate), physical activity (<4, ≥4 hours/week), BMI (≤24.9, 25–29.9, >29.9 kg/ m^2^), and duration of breast feeding (<1, 1–11, ≥12 months). Adjusted models included covariables that met criteria for a 10% change in the beta coefficient when modeling alcohol intake and mortality [6, 13, 14, 23]. We stratified adjusted models by age at baseline (<50, 50–64, ≥65 years), ethnicity (non-Hispanic white, Hispanic), cigarette smoking, and BMI to explore interaction. We performed the following sensitivity analyses: excluding those without pathologically confirmed invasive breast cancer (i.e. not including non-invasive lobular or in situ cancer), and excluding cases diagnosed five or more years before baseline (entry into the study).

We tested the proportionality assumption graphically based on the martingale residuals[24] and by including interactions of the predictors and the log of person time into the model[25]. When we found a violation of proportional hazards, which only occurred for age at baseline variable, we included a time-dependent variable of the non-proportional predictor in the model. All tests of statistical significance were two-sided and we considered *P* values < 0.05 as nominally statistically significant. All statistical analyses were conducted using SAS release 9.4 (SAS Institute, Cary, NC, USA).

## Results

Characteristics by alcohol intake are shown in Table 1. A total of 510 women reported regular alcohol use at some period in their life, while 606 women reported no regular alcohol use. Women who consumed >3 drinks/week tended to be non-Hispanic whites, with a lower BMI, higher level of education, higher level of physical activity and lower parity, and were more likely to have ever smoked or used oral contraceptives. Among the 510 who reported regular alcohol use and had information on consumption, the mean and median drinks per week were 6.4 and 5.1 and the range was 1.5 to 27.6 drinks per week. Mean follow-up time (years±SD) was 9.1± 4.1 years. During 10,076.8 person-years of follow-up, we observed 211 total deaths, of which 58 were breast cancer deaths and 153 were other non-breast cancer deaths.

**Table 1:** Average alcohol intake by demographic characteristics and lifestyle variables in a cohort of breast cancer survivors from the New York site of the Breast Cancer Family Registry.

n(%) or mean±SE^a^	Non-Drinkers (n = 606)	≤ 3 drinks/week (n = 155)	> 3 drinks/week (n = 355)	*p*-value
**Vital Status**				0.10
Alive	489 (80.7)	135 (87.1)	281 (79.2)	
Deceased	117 (19.3)	20 (12.9)	74 (20.9)	
*Breast cancer death*	34 (29.1)	5 (25.0)	19 (25.7)	
*Other death*	83 (70.9)	15 (75.0)	55 (74.3)	
**Age (years)**	55.13±0.58	54.28±1.04	54.53±0.74	0.71
**Age (years categorical)**				0.61
< 50	250 (41.3)	63 (40.7)	147 (41.4)	
50–64	208 (34.3)	56 (36.1)	136 (38.3)	
≥65	148 (24.4)	36 (23.2)	72 (20.3)	
**Ethnicity** ^**b**^				<0.0001
Non-Hispanic White	379 (62.5)	123 (79.4)	295 (83.1)	
Hispanic	126 (20.8)	18 (11.6)	36 (10.1)	
Other ^c^	96 (15.8)	14 (9.0)	22 (6.2)	
**Body mass index at baseline (kg/m**^**2**^**)** ^**b**^				<0.001
≤24.9	283 (46.7)	93 (60.0)	211 (59.4)	
25.0–29.9	202 (33.3)	39 (25.6)	104 (29.3)	
>29.9	119 (19.6)	22 (14.2)	40 (11.3)	
**Education** ^**a**^				<0.0001
Less than high school	69 (11.4)	7 (4.5)	13 (3.7)	
High school or vocational/tech	121 (20.0)	26 (16.8)	55 (15.5)	
College or graduate	414 (68.3)	122 (78.71)	287 (80.9)	
**Physical Activity (hours/week)** ^**d**^				<0.001
<4	367 (60.6)	86 (55.48)	167 (47.0)	
≥4	212 (35.0)	63 (40.65)	172 (48.5)	
**Age at menarche (years)** ^**b**^				0.75
<12	137 (22.6)	43 (27.74)	89 (23.9)	
12–13	328 (54.1)	82 (52.9)	193 (54.4)	
>13	132 (21.8)	29 (18.7)	76 (21.4)	
**Age at menarche (years)**	12.52±0.06	12.36±0.11	12.49±0.08	0.54
**Parity**	2.02±0.06	1.74±0.10	1.69±0.07	<0.001
**Age at first live birth (years)**	25.82±0.21	26.81±0.48	26.10±0.32	0.14
**Duration of breast feeding (months)**				0.49
<1	350 (57.8)	93 (60.0)	224 (63.1)	
1–11	163 (26.9)	36 (23.2)	84 (23.7)	
≥ 12	93 (15.4)	26 (16.8)	47 (13.2)	
**Menopause status** ^**e**^				0.42
Postmenopausal	394 (65.0)	106 (68.4)	245 (69.0)	
Premenopausal	205 (33.8)	46 (29.7)	108 (32.4)	
**Age at menopause (years)**	45.35±0.48	46.83±1.10	46.20±0.59	0.34
**Smoking history** ^**b**^				<0.0001
Never	387 (63.9)	73 (47.1)	128 (36.0)	
Ever	216 (35.6)	81 (52.3)	227 (63.9)	
**Hormonal birth control history** ^**b**^				<0.0001
Never	290 (47.6)	62 (40.0)	117 (32.0)	
Ever	308 (50.8)	93 (60.0)	237 (66.8)	
Period of time used (years)	4.73±0.26	4.42±0.43	5.62±0.35	0.05
**HRT history** ^**e**^				0.09
Never	504 (83.2)	127 (81.9)	280 (78.9)	
Ever	94 (15.5)	28 (18.1)	67 (18.9)	
Period of time used (years)	4.78±0.68	6.33±0.99	4.81±0.64	0.45
**Hormone Receptor Status**				0.78
*Cases diagnosed 1996 or earlier*				
ER-, PR-	24 (6.6)	5 (5.9)	16 (6.4)	
ER+, PR+	48 (13.2)	10 (11.8)	48 (19.3)	
ER+, PR-	10 (2.7)	0	10 (4.0)	
ER-, PR+	6 (1.6)	1 (1.2)	8 (3.2)	
Missing	277 (75.9)	68 (80.0)	167 (67.1)	
*Cases diagnosed after 1996*				
ER-, PR-	42 (17.4)	10 (14.3)	16 (15.1)	
ER+, PR+	67 (27.8)	24 (34.3)	30 (28.3)	
ER+, PR-	8 (3.3)	5 (7.1)	7 (6.6)	
ER-, PR+	15 (6.2)	3 (4.3)	5 (4.7)	
Missing	109 (45.2)	28 (40.0)	48 (45.3)	
**Tumor Stage**				
0	66 (10.9)	25 (16.1)	29 (8.2)	0.18
I	218 (36.0)	53 (34.2)	148 (41.7)	
II	48 (7.9)	12 (7.7)	27 (7.6)	
III	27 (4.5)	6 (3.9)	9 (2.5)	
IV	6 (1.0)	0	0	
Missing	241 (39.8)	59 (38.1)	142 (40.0)	

^a.^ Percentages may not add up to 100% due to missing

^b.^ ≤1% missing

^c.^ “Other” category includes the following race categories: African-American (3.4%), Asian (4.5%), Mixed race (2.9%), and Other races (1.1%).

^d.^ ≤3% missing

^e.^ ≤2% missing

Overall mortality, breast cancer-specific mortality, and non-breast cancer mortality were not significantly associated with alcohol consumption and these results were maintained after adjusting for confounders (Table 2). Specifically, compared to non-drinkers we found that both low and moderate to heavy levels of alcohol intake were not associated with greater overall mortality (≤3 drinks/week: HR^O^: 0.66, 95% CI: 0.38–1.14); > 3 drinks/week: HR^O^: 1.16, 95% CI: 0.85–1.58), breast cancer–specific mortality (≤ 3 drinks/week: HR^BC^:0.62, 95% CI: 0.19–2.03; >3 drinks/week: HR ^BC^: 0.96, 95% CI: 0.49–1.89), or non-breast cancer-specific mortality (≤3 drinks/week: HR ^NBC^: 0.73, 95% CI: 0.32–1.6; >3 drinks/week: HR^NBC^: 1.18, 95% CI: 0.75–1.86) after a mean of 9.1 years of follow-up in women affected with breast cancer at baseline.

**Table 2:** Association between alcohol consumption and overall, breast cancer- specific, and non-breast cancer mortality.

	No. of Subjects	Person years	No. of Deaths	Age adjusted HR (95% CI)	No. of Subjects	No. of Deaths	Multivariable HR (95% CI)
**Average Alcohol Use** ^**a**^							** **
*Overall Mortality* ^*b*^							
Non—drinkers	606	5138.1	117	Reference	580	116	Reference
≤3 drinks/week	155	1513.1	20	0.66 (0.41–1.05)	149	17	0.66 (0.38–1.14)
>3 drinks/week	355	3425.6	74	1.01 (0.77–1.33)	341	72	1.16 (0.85–1.58)
*Breast Cancer Specific Mortality* ^*c*^							
Non—drinkers	606	5138.1	34	Reference	474	25	Reference
≤3 drinks/week	155	1513.1	5	0.53 (0.21–1.36)	109	3	0.62 (0.19–2.03)
>3 drinks/week	355	3425.6	19	0.88 (0.50–1.54)	247	15	0.96 (0.49–1.89)
*Non-Breast Cancer Mortality* ^*d*^							
Non—drinkers	606	5138.1	83	Reference	436	60	Reference
≤3 drinks/week	155	1513.1	15	0.72 (0.42–1.23)	113	8	0.73 (0.32–1.60)
>3 drinks/week	355	3425.6	55	1.06 (0.76–1.46)	245	30	1.18 (0.75–1.86)

^a^ Defined as total reported alcohol intake from age 12 until age at baseline

^b^ Adjusted for age at baseline, BMI, ethnicity, menopausal status, age at menarche, hormone replacement therapy use (HRT), and cigarette smoking.

^c^ Adjusted by age at baseline, BMI, ethnicity, physical activity (PE), age at first live birth (AAFLB), menopause status, oral contraceptive use (OC), education level, breast feeding duration, and cigarette smoking.

^d^ Adjusted by age at baseline, BMI, ethnicity, menopausal status, age at menopause, HRT, and cigarette smoking.

We also examined the association of alcohol consumption and overall, breast cancer- specific, and non-breast cancer mortality using light drinkers (≤ 3 drinks/week) as the referent category instead of never drinkers. Compared to light drinkers, we found a non-significant elevated risk of overall, breast cancer- specific, and non-breast cancer mortality for both non-drinkers and heavy drinkers (>3 drinks/week). Compared to light drinkers, women who reported never drinking had a 52% increased risk for overall mortality (HR^O^: 1.52, 95% CI: 0.95–2.42), an 89% increased risk for breast cancer-specific mortality (HR^BC^: 1.89, 95% CI: 0.74–4.86), and 40% increased risk for non-breast cancer mortality (HR^NBC^: 1.40, 95% CI: 0.81–2.4). We observed the same general pattern for heavy drinkers (> 3 drinks/week). When compared to light drinkers, heavy drinkers had a 54% increased risk for overall mortality (HR^O^: 1.54, 95% CI: 0.96–2.47), and 66% increased risk for breast cancer-specific mortality (HR^BC^: 1.66, 95% CI: 0.62–4.45), and 48% increased risk for non-breast cancer mortality (HR^NBC^: 1.48, 95% CI: 0.85–2.56).

**Fig 1:**
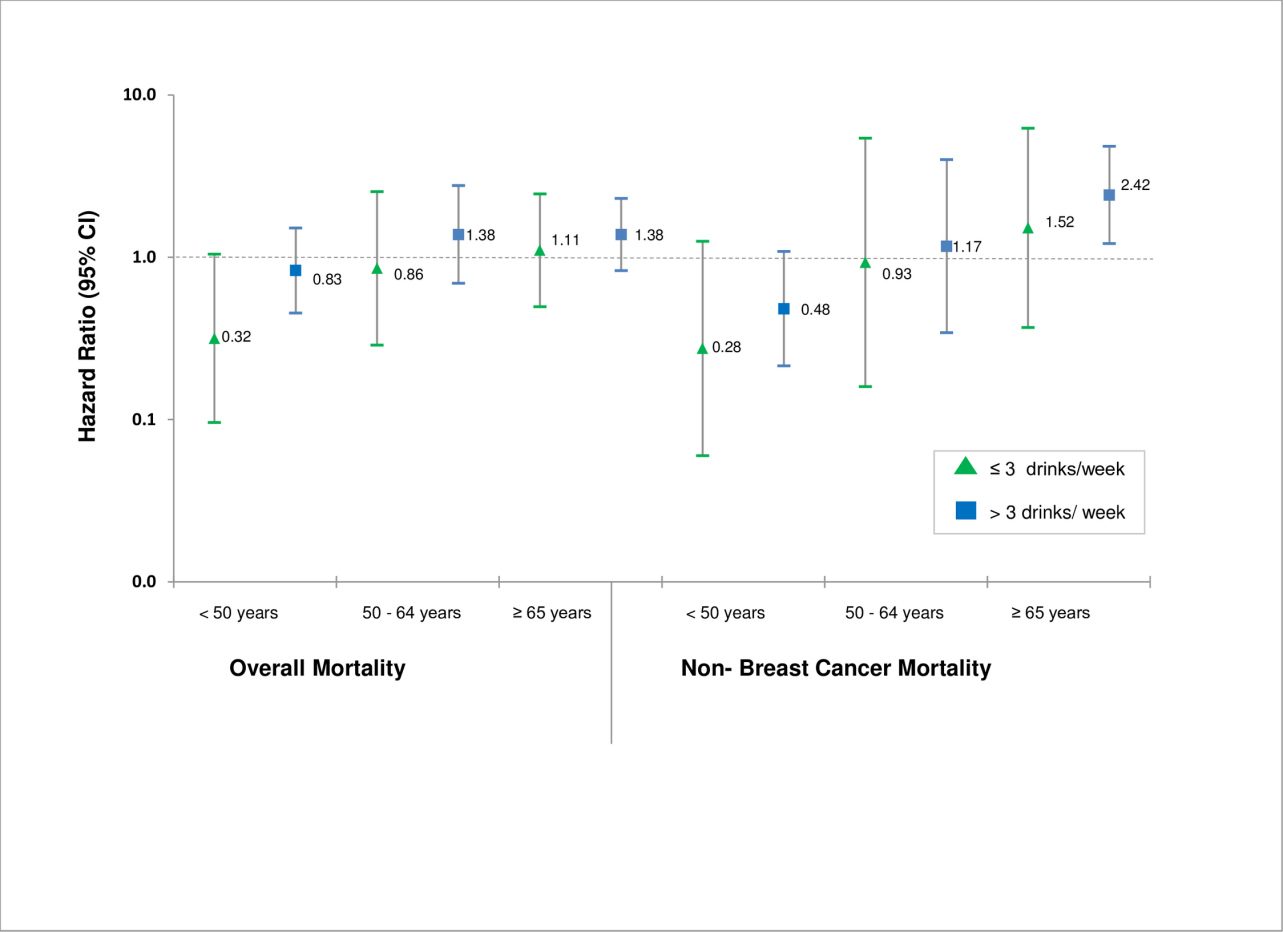
Adjusted log hazard ratios and 95% confidence intervals for overall and non-breast cancer mortality in breast cancer survivors by age at baseline.

We examined the association of alcohol consumption and overall, breast-cancer specific, and non-breast cancer mortality stratified by age category (<50, 50–64 and ≥65 years), and did not observe any major differences with the exception of women ≥65 years. Stratification by age at baseline showed that low and moderate alcohol consumption was protective in women <50 years, although none of these findings were statistically significant. For women younger than 50 years of age at baseline with low alcohol consumption (≤ 3 drinks/week), we observed a nearly 70% reduction in risk of overall mortality that was borderline significant after adjusting for confounders, HR^<50^: 0.32, 95% CI: 0.10–1.05 (Fig 1) In contrast, compared to non-drinkers, women ≥65 years of age who consumed >3 drinks/week had non-statistically significant 30% increased risk for overall mortality (HR^O^: 1.38, 95% CI: 0.83–2.30) and a significant two-fold increase in risk for non-breast cancer mortality (HR^NBC^: 2.42, 95% CI: 1.22–4.82) after adjusting for confounders (Fig 1).

Stratification by cigarette smoking status showed that moderate to heavy drinking was associated with an increased risk for overall and non-breast cancer mortality in women who reported ever smoking, but not in never smokers, although none of these findings were statistically significant. Compared to non-drinkers, women who reported smoking and who consumed > 3 drinks/week had a 24% increased risk for overall mortality (HR^O^: 1.24, 95% CI: 0.85–1.80) and a 34% increased risk for non-breast cancer mortality (HR^NBC^: 1.34, 95% CI: 0.85–2.1). Effect of alcohol intake on rate of mortality did not differ when stratifying by ethnicity (non-Hispanic whites and Hispanics), BMI (normal and overweight/obese), or by ER/PR hormone receptor status (results not shown).

The results were also similar when limiting models to invasive breast cancer (excluding carcinoma in-situ cases). Specifically, compared to non-drinkers we found that both low and moderate to heavy levels of alcohol intake were not associated with greater overall mortality (≤3 drinks/week: HR^O^: 0.66, 95% CI: 0.41–1.07); > 3 drinks/week: HR^O^: 1.01, 95% CI: 0.76–1.35), breast cancer–specific mortality (≤ 3 drinks/week: HR^BC^:0.56, 95% CI: 0.22–1.45; >3 drinks/week: HR^BC^: 0.87, 95% CI: 0.49–1.55), or non-breast cancer specific breast cancer-specific mortality (≤3 drinks/week: HR^NBC^: 0.70, 95% CI: 0.40–1.23; >3 drinks/week: HR^NBC^: 1.06, 95% CI: 0.75–1.48) in women affected with invasive breast cancer at baseline. Results were also similar from models limited to cases diagnosed within five years of baseline (results not shown).

## Discussion

In this family-based prospective cohort study of women with breast cancer, overall mortality, breast cancer–specific, and non-breast cancer mortality was not significantly associated with average alcohol consumption. Our findings are consistent with several prospective studies (median follow-up time 7.4–12.6 years) which observed no association with increased overall and breast cancer-specific mortality and alcohol consumption [11, 16, 26, 27]. However, of note is the heterogeneity between studies of how alcohol was defined since each of these studies evalauted alcohol consumption at different time frames and using different levels of intensity.Two of these prospective studies evaluated consumption of alcohol both before and after breast cancer diagnosis, and both found no increased associations with mortality at all levels of alcohol consumed when compared to non-drinkers for post-diagnosstic alcohol intake[11, 27]. This finding of no associatoin with post-diagnostic alochol consumption and mortality is consistent with a report from the After Breast Cancer Pooling Project, the largest and longest study to date assessing post-diagnostic alcohol consumption and survival and with a recent meta-analysis of post-diagnostic alcohol consumption [26, 28].

To our knowledge, only two other studies assessed cumulative alcohol intake and breast cancer–specific mortality, the European Prospective Investigation into Cancer and nutrition (EPIC) prospective cohort study and a large Danish prospective cohort, the ‘Diet, Cancer and Health’ (DCH) study[14, 29]. In EPIC, alcohol consumption was assessed through self-reported weekly consumption of wine, beer and liquor at ages 20, 30, 40, 50 years and categories of average alcohol use were calculated as follows: never drinkers, 1–4.9 g/day (reference category), 5–14.9, 15–29.9, 30–59.9, and ≥60 g/day. Compared to light drinkers, they found a non-significant increased risk of breast cancer–specific mortality for never drinkers (HR: 1.31, 95% CI: 0.97–1.76) and for every level of alcohol consumption, with increased risks ranging from 17%-20% [29]. Although we did not define our categories of alcohol consumption identically, the EPIC findings are consistent with our finding of a non-significant increased risk for breast cancer-specific mortality for both non-drinkers and heavy drinkers compared to light drinkers. The DCH study assessed cumulative alcohol intake from age 20 until one year before baseline and calculated drink years (dy) as one unit of alcohol/day in one year, which was examined both as a linear and as a categorical variable (0–10 dy (reference), 10–40 dy, >40 dy) [14]. They did not report any significant associations with breast-cancer specific mortality, although they did find a non-statistically significant 27% increased risk for breast cancer–specific mortality for women in the highest drink year category (>40 dy) after adjusting for clinical prognostic factors (node, grade, tumor size and receptor status) as well as other covariates [14].

With respect to pre-diagnostic alcohol intake, Newcomb et al reported a non-linear relationship with increasing pre-diagnostic alcohol consumption and survival. Compared to non-drinkers, improved survival was observed for moderate drinkers (3–6 drinks/week) and no association was observed for heavier drinkers (≥ 10 drinks/week)[11]. This U-shaped pattern was also suggested in a report by Hellman et al for pre-diagnositic alcohol intake although none of the reported findings were statistically signficant[16]. Conversely, at least two studies have observed a positive association with mortality, particularly for higher intakes (e.g., 6% increase, 95% CI 1.03–1.10 when intake was >20g/day) [7, 8].

The mechanism for how alcohol intake, particularly before diagnosis, could influence mortality in women diagnosed with breast cancer is unclear. Alcohol consumption is believed to increase breast cancer risk through increased endogenous estrogen and androgen hormone levels and increased DNA damage to the mammary tissue. Thus reducing the amount of estrogen is important for controlling disease severity and progression, and ultimately breast cancer-specific mortality. However, pre-diagnostic alcohol consumption has been associated with reduced odds of having a tumor with high tumor necrosis levels and p53-positive tumors, both of which are associated with worse prognosis and increased mortality[30]. Additionally, moderate alcohol consumption has been demonstrated to have cardio-protective effects and can have a favorable impact on all-cause mortality [15].

Alcohol intake has been shown to affect receptor status [31], which itself is associated with breast cancer mortality[32]. While our finding that the relation between alcohol intake and mortality in women affected with breast cancer was not modified by ER/PR receptor status is consistent with previous studies, this result was limited by missing receptor status data and should be interpreted with caution [8, 12, 14, 30].

Our results suggest that affected women ≥65 years, with a family history of breast cancer who consume >3 drinks/week are at a greater risk of non-breast cancer mortality. It is important to note that we did not have information on comorbidities, such as diabetes or cardiovascular disease, and can therefore not rule out confounding by comorbidities. Of the prior studies that examined alcohol consumption and mortality, only one study of post-diagnosis alcohol consumption by Kwan et al [26] adjusted for comorbidities (a history of either diabetes, hypertension, myocardial infarction (MI) and/or stroke) and found no association with any level of alcohol intake and overall or breast-cancer specific mortality. While that analysis did not stratify by age category, they did stratify by menopausal status and observed similar non-significant results. Other studies have not observed stronger associations in older women [7, 9, 10, 16] although one study showed an increased risk of recurrence in post-menopausal women as compared to premenopausal women (HR:1.51, 95% CI: 1.05–2.19) [12, 26].

A key limitation of our study is the inability to distinguish between pre-diagnostic and post-diagnostic alcohol consumption due to the method with which we collected information on alcohol. Also of note is that long-term alcohol consumption was assessed retrospectively. Additionally, we did not have information on the changes in alcohol consumption following a diagnosis. However, several reports have noted that alcohol consumption patterns do not change following a breast cancer diagnosis [10, 11, 20–22]. Although we collected detailed information at different periods of life prior to the outcome (death), we cannot rule out errors in recall of alcohol consumption during different age periods and potential misclassification of alcohol exposure. Additionally, stigma associated with heavier drinking patterns could also lead to under-reporting of the amount of alcohol consumed. As mentioned earlier, while we had adequate information on potential confounders including physical activity, BMI, smoking, and menopausal status, we did not have information on comorbidities including cardiovascular disease and could not account for them in the analyses. We also could not adjust for tumor stage, due to missing data and limited variability in our sample in which most women with staging were stage I. Finally, although mean follow-up time was over 9 years, our lack of association could be due to insufficiently long follow-up time. A longer follow-up could have allowed us to observe more events, particularly breast cancer–specific deaths.

A strength of this study was that the population explored was a diverse high-risk family based cohort with very little missing data on alcohol consumption, and use of linkage with the NDI for mortality outcomes. Family reported deaths and participants who met NDI recommended cutoffs for being an accurate match were defined as deceased[19].

Risk and benefit considerations are important when taking into account the opposing relation between moderate alcohol intake and cardiovascular disease risk versus alcohol intake and breast cancer risk. This may be especially of concern to women who have both a personal history of breast cancer diagnosis as well as a family history of breast cancer[33], and who look for recommendations to minimize their risk of recurrence or rate of mortality[34]. While much of the current evidence does not suggest that alcohol consumption in high risk women increases mortality from breast cancer, most studies adjust for family history rather considering it as an effect modifier[6, 7, 10, 11, 15], with the exception of one [7], and our results are consistent with this study.

In conclusion, for women with breast cancer from a high risk family, alcohol intake is not associated with overall, breast cancer, or non-breast cancer mortality exclusive of women ≥65 years who consumed >3 drinks/week.

## Abbreviations

BCFR: Breast Cancer Family Registry
NDI: National Death Index
HRT: Hormone replacement therapy
BMI: Body mass index
ER: Estrogen receptor
PR: Progesterone receptor
SE: Standard error
SD: Standard Deviation
HR: Hazard ratio
CI: Confidence interval
